# Effects of Iron and Zinc Biofortified Foods on Gut Microbiota In Vivo (*Gallus gallus*): A Systematic Review

**DOI:** 10.3390/nu13010189

**Published:** 2021-01-09

**Authors:** Mariana Juste Contin Gomes, Hércia Stampini Duarte Martino, Elad Tako

**Affiliations:** 1 Department of Nutrition and Health, Federal University of Viçosa, Viçosa, MG 36570-000, Brazil; mariana.juste@hotmail.com (M.J.C.G.); hercia72@gmail.com (H.S.D.M.); 2 Department of Food Science, Cornell University, Stocking Hall, Ithaca, NY 14850, USA

**Keywords:** zinc, iron, minerals, short chain fatty acids, intestinal health, bacteria taxa, diversity analysis

## Abstract

Dietary iron and zinc deficiencies are a global health concern. Bacteria that colonize the gastrointestinal tract depend on minerals to maintain their activities; thus, recent evidence suggests that biofortified foods can modulate the host’s beneficial bacterial taxa. The current review analyzed the research data that linked between iron and zinc biofortified foods and gut microbiota modulation. The data analysis was based on the PRISMA guidelines and the data search was performed at PubMed, Web of Science, Science Direct, and Scopus databases for experimental studies published from January 2010 until December 2020. The five selected studies were conducted in an experimental in vivo model (*Gallus gallus*). The identified and discussed research showed positive effects of biofortified foods on the composition and function of the gut microbiota. Further, an increase in short chain fatty acids producing bacterial populations as *Lactobacillus* and *Ruminococcus*, and a decrease in potentially pathogenic bacteria as *Streptococcus*, *Escherichia*, and *Enterobacter* was identified due to the consumption of biofortified foods. In conclusion, biofortified foods may contribute to improved gut health without increasing the colonization of pathogenic bacteria. The dietary inclusion of approximately 50% of iron/zinc biofortified foods has a significant beneficial effect on the gut microbiota. Additional studies in humans and animal models are warranted to further establish the suggested effects on the intestinal microbiome. PROSPERO (CRD42020184221).

## 1. Introduction

Dietary deficiencies of vitamins and minerals such as iron (Fe), zinc (Zn), vitamin A, iodine, and folic acid are common health concerns worldwide, with significant physiological and developmental consequences. Globally, Fe deficiency is the most widespread nutritional disorder that affects approximately two billion people [1]. Zinc is the second highest micronutrient deficiency, affecting approximately 17% of the global population [2]. As a part of the battle aimed at decreasing the dietary prevalence of micronutrient deficiencies, several governmental programs have been established, and strategies were developed in order to effectively produce Fe and Zn biofortified and fortified foods.

Biofortification is the process of conventionally breeding staple food crops that are rich in micronutrients, such as vitamin A, Zn, and Fe. Biofortification is a food-based approach that aims to improve the nutritional value of staple foods that are consumed by the relevant target populations that are most affected by a specific nutritional deficiency with potential or existing malnutrition [3]. Previous studies indicated that the consumption of biofortified foods such as common beans, rice, sweet potatoes, and pumpkins increased the dietary micronutrients delivery and intake, and therefore, improved the physiological status and overall health of the population [4,5,6,7]. Linked to the delivery of a greater amount of the target nutrient and as part of the biofortification process, the foods chosen by the biofortification programs are also rich in other compounds which may affect overall health. The food matrix of biofortified foods can have a differential effect on the gut microbiota and can modulate the bacterial taxa in the colon. In this review, we present the effects of biofortified foods with micronutrients or their fractions such as flour or soluble extracts on gut microbiota in vivo, and only animal model studies that had an appropriate control group, composed by the test food or its conventional/non-biofortified fraction, were included. Common beans, for example, are targets for biofortification due to their multiple beneficial health effects, such as anti-inflammatory, antioxidative, and ability to reduce the risk of cardiovascular diseases in vivo [8,9], with further anti-inflammatory, antihyperlipidemic, and antihypertensive properties as demonstrated in vitro [10,11]. In addition, several governmental programs have focused on food fortification, which take place on an industrial scale and aimed to improve the amount of micronutrients consumed via basic foods and food products [12]. 

It was previously established that the human gastrointestinal tract inhabits a diverse and complex microbiota, composed of trillions of microorganisms that are distributed along the intestine in a symbiotic relationship with its host [13]. The abundance of this microbiome is modulated by several external and internal factors, amongst them, are the subject’s dietary habits and composition [14]. Previously, the scientific literature defined a “healthy gut microbial profile” that is composed of short chain fatty acids (SCFA) producing bacteria that benefits the host by regulating the intestinal homeostasis, contributing to the absorption of minerals [15] via the efficient functionality of the duodenal brush border membrane (BBM). In addition, a beneficial bacterial taxa profile was suggested to reduce the incidence of preneoplastic lesions and tumors in vivo, and ability to delay the progression of cancer associated with inflammatory bowel disease [16]. 

Experimental studies that evaluated the effects of micronutrient fortified foods on the gut microbiota are scarce [17,18]. There are some clinical studies that evaluate these effects, however, these were conducted with toddler populations [19,20,21], that present a still forming eating patterns and in constant change. In addition, the frequent use of antibiotics may lead these populations to not have a well-established resident intestinal microbiota [22,23]. In contrast, the role of dietary biofortified foods and how it may affect the composition and function of the gut microbiome has been recently investigated. Despite the available knowledge on the effects of biofortified foods on dietary mineral bioavailability, there is no evidence that increased concentrations of dietary minerals and as part of a complete meal (containing dietary fibers, proteins, lipids) has a beneficial effect on gut microbiota in vivo. 

Therefore, the objective of the current study was to systematically review the experimental studies that evaluated the effects of the consumption of Fe and Zn biofortified foods or their derivatives, such as flour or soluble extracts, on the gut microbiota. Hence, if the current review provides evidence that Fe and Zn biofortified foods have a beneficial effect on the gut microbiota, we suggest further increased dietary consumption of Fe and Zn biofortified foods by populations with these micronutrient deficiencies. 

## 2. Materials and Methods 

### 2.1. Protocol and Registration

This systematic review was carried out in accordance with the Preferred Reporting Items for Systematic review and Meta-Analysis (PRISMA) protocols [24] and registered in PROSPERO (CRD42020184221). The research question to be reviewed was: “What are the effects of the consumption of biofortified foods with some micronutrient on the gut microbiota of in vivo models”? This is the first study to review the effects of biofortified foods on gut microbiota. 

### 2.2. Literature Search

Two researchers independently searched for original articles. The search was carried out in PubMed, Web of Science, Science Direct, and Scopus databases for experimental studies conducted in animal models that evaluated the effects of biofortified foods on the gut microbiota. Filters were used to select articles published from January 2010 until December 2020. The last search date was 4 December 2020.

The descriptors were identified based on Medical Subject Headings (MeSH) and the following search strategy was designed and utilized: (“Microbial Profile” OR “Cecum Microbiome” OR “Gastrointestinal Microbiome” OR “Gastric Microbiome” OR “Gut Microbiome” OR “Gut Microbiomes” OR “Gut Microflora” OR “Gut Microbiota” OR “Gut Microbiotas” OR “Gastrointestinal Flora” OR “Gut Flora” OR “Gastrointestinal Microbiota” OR “Gastrointestinal Microbiotas” OR “Gastrointestinal Microbial Community” OR “Gastrointestinal Microflora” OR “Intestinal Microbiome” OR “Intestinal Microbiomes” OR “Intestinal Microbiota” OR “Intestinal Microbiotas” OR “Intestinal Microflora” OR “Intestinal Flora” OR “Microbial Populations” OR “Enteric Bacteria” AND Biofortification OR Biofortified OR “food biofortification” OR “foods biofortification” OR “Biofortified Foods” OR “Biofortified Food” OR “Biofortified Crops” OR “Biofortified Crop”). The logical operators “AND” or “OR” were used to combine the descriptors. 

### 2.3. Screening and Eligibility of Records

The eligibility criteria were formulated with reference to participants, interventions, comparisons, outcomes, and study design (PICOS). Duplicate studies were excluded and, the search and screening for titles and abstracts were carried out independently by the authors according to the inclusion and exclusion criteria (Table 1). After screening, in vitro studies, reviews, consensus papers, letters to editor, books, book chapters, theses, dissertations, and non- animal studies were excluded, and studies with biofortified foods that evaluated the gut microbiota were selected.

The potentially eligible research articles were read in full independently by authors and assessed for compliance with the established eligibility criteria. Discrepancies between reviewers were resolved through consensus with a third reviewer and the reference lists of the studies included were hand searched to identify other relevant trials. If the data were not reported or unclear, we directly contacted authors via e-mail.

### 2.4. Data Extraction

After reading and reviewing the selected research articles in full, the data were compared to ensure integrity and reliability. Divergent decisions were resolved by consensus. For each experimental study included, we extracted relevant information related to the authors, publication year and experimental model features as species, sex and age. To access the research methods, we extracted specific information related to the experimental groups, number of animals per group, type of food intervention and method of consumption of the intervention. For the control of test food intake, we extracted information related to the type of biofortified food that was used in the intervention, the type of micronutrient incorporated in the food, the duration of the intervention, the methods of evaluation of the gut microbiota and the main results. 

For this review, data from the eligible studies are expressed in tables and figures. We provided a narrative synthesis of the results according to the main characteristics and results related to the topic addressed.

### 2.5. Risk-Of-Bias Assessment

The methodological quality of the included studies was assessed and the risk of bias was verified using the Systematic Review Centre for Laboratory Animal Experimentation Risk of Bias (SYRCLE RoB) tool [25], which is responsible for identifying the study quality and to measure the bias in research involving animal studies [26]. The SYRCLE RoB toll considers 10 entries that are related to six types of bias: selection bias, performance bias, detection bias, attrition bias, reporting bias, and other. For each included study, the six bias types were classified as “high” (+), “low” (–), or “unclear” (?).

To improve the quality evaluation of the included studies in this review, the criteria set forth in the Animal in Research: Reporting in Vivo Experiments (ARRIVE) guidelines [27] were used. A checklist of 20 items that evaluate essential descriptions about the experimental model, number of animals, study design, allocation of animals to experimental groups, methodological basis, statistical draw, and result measures were evaluated. For each criterion was filled out “0” for “not reported” or “1” for “reported”. The final score was displayed as a percentage for better visualization of the study quality. 

## 3. Results

### 3.1. Selected Studies

**Figure 1:**
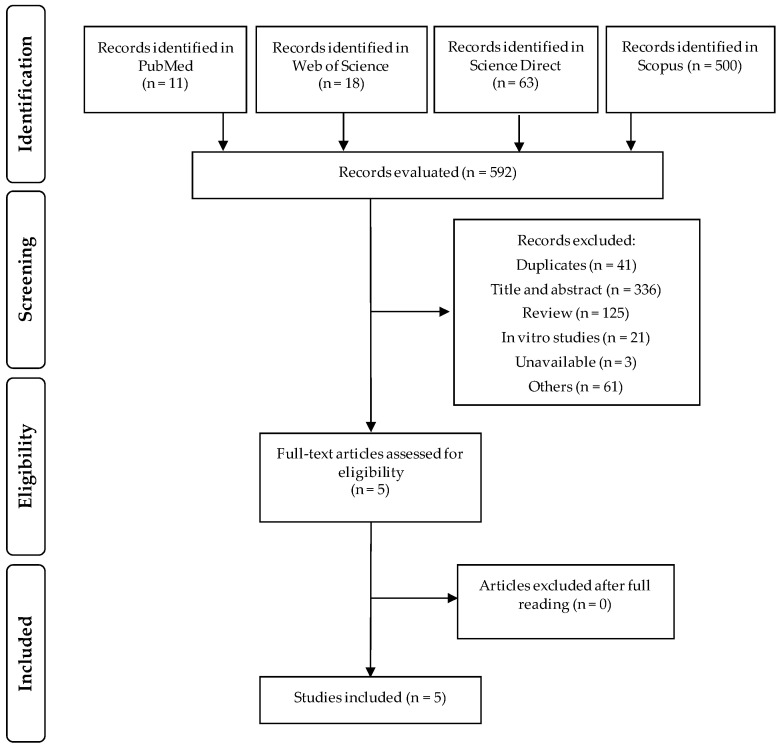
Flow diagram of the literature search process.

The flow diagram with the number of selected or excluded articles in each selection step was built in accordance with PRISMA guidelines (Figure 1). Altogether, 592 articles were identified in the PubMed (*n* = 11), Web of Science (*n* = 18), Science Direct (*n* = 63), and Scopus (*n* = 500). Of these, 587 articles were excluded: duplicate studies (*n* = 41), title, abstract and articles that were not suited to the topic (*n* = 336), review articles (*n* = 125), in vitro studies (*n* = 21), studies that could not be accessed (*n* = 3), and others scientific materials such as books, book chapters, or encyclopedia (*n* = 61). The remaining five articles were selected and after reading in full, all of them were eligible for this review. With the search in the reference lists we did not identify other relevant studies. All included studies were published from January 2010 and until December 2020.

### 3.2. Characteristics of the Included Studies 

The five original papers selected and included in the present systematic review were performed in the United States and the experimental model that was used was Cornish Cross broiler (*Gallus gallus*) [15,28,29,30,31]. Four studies used *Gallus gallus* hatchlings, male and female starting at day of hatch [15,28,30,31], and two studies performed the experiment at the embryonic stage (*Gallus gallus*) [28,29] (Table 2).

The studies were based on the consumption of biofortified foods with micronutrients. Three studies evaluated Fe biofortified carioca beans (*Phaseolus vulgaris* L.) [15,29,30], one study evaluated Fe biofortified wheat (*Triticum aestivum*) [28], and one evaluated Zn biofortified wheat [31]. Other details of the characteristics of the eligible studies were included and described in Table 2.

All included studies compared the biofortified food with the parallel standard food. The offered dosage varied according to the specific study and all reviewed studies were different. Reed et al. [31] tested 75% of Zn biofortified wheat in the diet, totaling 46.5 μg Zn/g; Reed et al. [30] tested 34.6% of Fe biofortified bean in the diet, totaling 48.7 μg Fe/g; Dias et al. [15] tested 42% of Fe biofortified bean in the diet, totaling 47.04 μg Fe/g; Dias et al. [29] performed utilized the intra amniotic administration (*in ovo* feeding) in vivo approach and assessed the effects of 50 mg/mL of Fe biofortified bean soluble extract per egg; and Beasley et al. [28] tested in the first study (via *intra amniotic administration*) 50 mg/mL of soluble extract from Fe biofortified wheat, and in a consecutive study evaluated 80% of Fe biofortified wheat in the diet, totaling 28.9 μg Fe/g.

### 3.3. Main Findings

The reviewed experimental studies demonstrated that Fe and Zn biofortified foods provide several health benefits to the host and improved the intestinal bacterial profile that leads to a healthier gut.

Of the five studies evaluated, three studies that performed the 16S rRNA gene sequencing reported significant differences in β-diversity between biofortified food vs. control treatment groups [15,28,31]. Two studies reported a significant increase in *Firmicutes* [15,30] and one study reported a reduction of the *Firmicutes* phyla [28]. Only one study reported a significant increase in the *Bacteroidetes* phyla after the consumption of the assessed biofortified food [31] (Table 3). Positive findings that are associated with SCFA producing bacteria were shown in the majority of the reviewed studies. These findings include an increased abundance of lactic acid bacteria [31], butyrate producing bacteria [30], and a general increased abundance of beneficial SCFA producing bacteria [15], that leads to an increase in SCFA production (acetic, propionic and valeric acids). Further, an increase in *Bifidobacterium* and *Lactobacillus* probiotic genera abundance in the Fe biofortified group was observed [28], and two studies reported an increase in bacteria linked to phenolic catabolism in the group that was fed the Fe biofortified material based diet [15,31].

In related to risk of bias, all studies included have described their titles and abstracts properly, presented the primary and secondary objectives in the manuscript introduction section, provided an ethical statement, included an adequate experimental protocol, and other relevant details in the manuscript methods section, and they all showed the dose of biofortified food offered to the animals. All studies described the route of consumption of biofortified food that was offered to the animals and all studies provided the information on how the biofortified foods were obtained and treated before use or provide the reference of a scientific article with the appropriate information and relevant methodologies.

**Figure 2:**
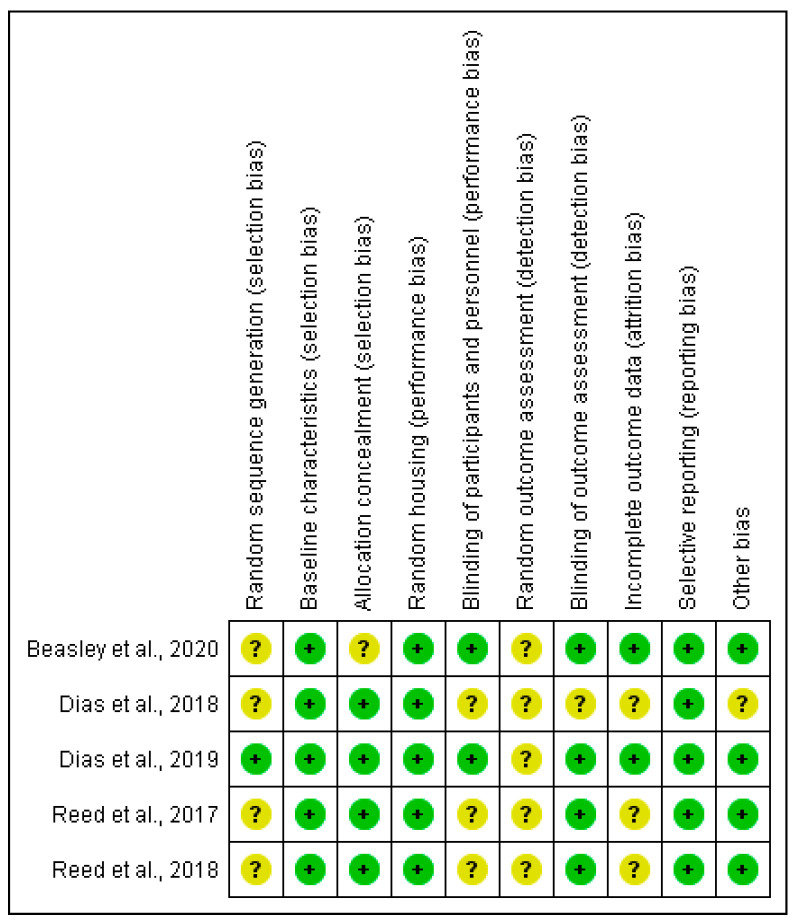
Risk of bias summary: review authors’ judgments about each risk of bias item for each included study. +, low risk; ?, unclear.

Figure 2 summarizes the risk of bias that is related to each manuscript that was included in this review and the ARRIVE guideline was summarized in the Supplementary Material (Table S1). Due to the nature of studies included, the risk categorization is not specified. The random sequence generation, the random of the outcome assessment, and the blinding of the outcome assessor were the most uncertain points detected in the manuscripts included in this review. The randomness of outcomes improves the quality of the selection and measurement bias and it is advisable to follow a methodology of randomization and blind evaluation of the results [32]. 

## 4. Discussion

In recent years, there has been an increase in the overall consumption of biofortified foods and in the development of new strategies related to food biofortification, aiming to increase the supply of nutrients, minerals and vitamins to populations with higher risk of dietary deficiency. Hidden hunger is characterized by inadequate intake of one or more micronutrients such as pro-vitamin A, Fe, and Zn. These types of nutritional deficiencies install imperceptibly in the body and lead to serious complications, especially for most vulnerable groups, as children of preschool age, pregnant women, lactating women and the elderly [33]. Food fortification may be an effective strategy, but it may not be sustainable, and may not reach all relevant target populations. Biofortification, on the other hand, can be a more efficient, economical, and sustainable alternative to maintain long-term nutrient consumption and improve the health of relevant populations with poor access to balanced diet.

### 4.1. Impact of Fe and Zn Biofortification on the Gut Microbiota In Vivo

In recent years, several studies were designed and conducted to assess the efficacy of biofortified foods, also aiming to evaluate productivity, in an economic context, and the consumption to these foods by the population [34]. This includes assessing the dietary mineral bioavailability and gene expression of BBM proteins that are associated with mineral absorption [35], including the effects of the tested dietary composition on intestinal functionality and host’s intestinal microbial taxa composition. However, there is still no consensus whether the increased micronutrients content in foods influences the gut microbiota. In this review, we provided evidence that the consumption of Fe and Zn biofortified foods may improve the host’s gut microbiota composition and function.

All studies included in this review utilized the established *Gallus gallus* model, which has been used to assess the bioavailability of minerals, specifically Fe and Zn, as this model exhibits the appropriate responses to Fe and Zn deficiencies and can serve as a model for Fe and Zn dietary bioavailability and absorption [36,37,38]. The *Gallus gallus* model inhabits a dynamic and complex intestinal microbiota, similar to that of humans, with predominance of the *Firmicutes*, *Proteobacteria* and *Actinobacteria* phyla [15,30,39]. In addition, there is a great homology (>85%) between human and chicken intestinal genes responsible for the expression of BBM proteins involved with the Fe and Zn absorption, such as Divalent Metal Transporter 1 (DMT1), Duodenal cytochrome b (DcytB), Zinc Transporter 1 (ZnT1) and Ferroportin (FPN) [40]. The 16S rRNA gene sequencing is a specific method for studying bacterial phylogeny and taxonomy and has been widely used in studies that evaluate human and animal microbiome. The 16S rRNA gene has been preserved for generations, it is present in most bacteria, allowing precise investigation in the field of the microbiome and it allows stratification at the genus and species level [41]. In this review, some studies used the linear discriminant analysis effect size (LEfSe) [42] to investigate significant bacterial biomarkers that could identify differences in the gut microbiota of treatment groups. The four studies that performed the 16S rRNA gene sequencing reported a change in β-diversity between treatment vs. control groups [15,28,31]. Changes in the β-diversity can occur in an experimental group after treatment, but do not necessarily indicate a beneficial variation in the bacterial taxa. Generally, the response of the microbial taxa to the consumption of Fe and Zn biofortified foods varied in terms of taxonomic abundance but show a similar pattern in qualitative terms.

Zinc is an essential mineral with catalytic, structural and regulatory functions that has a refined homeostatic control, making it difficult to identify its inadequate levels in the body [43]. Bacteria that colonize the gastrointestinal tract are dependent on minerals, and the bacterial activity contribute to minerals solubility, making Zn biofortified foods a promising strategy to improve intestinal health, and potentially via bacterial fermentation activities. Reed et al. [31] observed an increase in *Lactobacillus reuteri*, members of *Dorea*, *Clostridiales*, *Ruminococcus*, and *Lachnospiraceae* in the group that received Zn biofortified wheat based diet. As Zn is essential for bacteria, the abundance of Zn-dependent microorganisms is up regulated in an environment with higher Zn bioavailability [44], this includes the *Ruminococcus* genus, that houses species of gram-positive bacteria that degrade cellulose or polysaccharides of the diet, especially resistant starch. The fermentation activity of SCFA-producing bacterial populations is recognized as an important contributor to the overall health of the gut ecosystem [45]. In addition, the abundance of *Lactobacillus reuteri* in the biofortified group suggests that the wheat-based diet provides prebiotic properties, with potential modulation and beneficial effect on the host’s intestinal bacterial profile, since *L. reuteri* interacts with both epithelial and non-epithelial cells with potent anti-inflammatory effects [46,47]. Further, the *Clostridiales* order has also shown an increased abundance in the biofortified group. *Clostridiales* belongs to the *Firmicutes* phylum, and is represented by fermenting microorganisms and SCFA producers in the intestine, mainly butyrate, which may lead to improvement in the host’s gut health [48,49]. The increase in SCFA-producing bacteria, specifically butyrate producers in the Fe biofortified group was also documented [30].

The gut microbiota is shaped directly by the host’s dietary habits and the presence of plant-origin dietary ingredients that modulate the colonization of selective bacterial populations. It was recently shown that in addition to the known plant primary metabolites, as soluble and insoluble dietary fibers, proteins and carbohydrates, the secondary metabolites, specifically phytochemicals, as phenolic compounds, terpenoids, and alkaloids, can also have antimicrobial properties, which can modify the composition and function of the intestinal microbiota [50]. The selective modulation of intestinal microorganisms that arises when consuming a certain food, occurs in conjunction with the process of metabolizing the components that are present in it. This allows the host to absorb and transform these phytochemicals and supply the required metabolites to the relevant bacterial populations. The presence of phenolic compounds and the highest content of Fe in biofortified beans [30] beneficially modulated the abundance of bacteria involved in phenolic catabolism, such as *Faecalibacterium prausnitzii*, *Enterococcus* spp., *Barnesiella* spp., and members of *Dehalobacteriaceae* and it does not seem to affect the composition nor genetic capacity of the gut microbiota [30].

This same qualitative pattern of the gut microbial taxa following the consumption of biofortified foods based diets was also shown in studies by Dias et al. [15,29]. An acute exposure study that evaluated the effects of Fe biofortified bean soluble extracts on the gut microbiota, observed a reduction in the relative abundance of pathogenic bacteria such as *Escherichia coli* and *Clostridium* [29]. In the long term in vivo feeding trial (*Gallus gallus*), the authors observed a predominance of SCFA-producing *Firmicutes* [15]. In addition, the authors found a greater abundance of *Eggerthella lenta* in the biofortified group. *E. lenta* is an intestinal bacterium belonging to the *Actinobacteria* phylum. These species have the ability to convert catechin and epicatechin into their metabolic derivatives, with potential to increase the bioavailability of catechin metabolites and improve intestinal health [51]. The greater abundance of *Coriobacteriaceae*, specifically *Eggerthella lenta* and *Lachnospiraceae*, butyrate producers [52], in the Fe biofortified bean group indicates on the protective potential of the biofortified foods to the host and via microbial activity, and without adverse changes in the microbiome’s composition.

Wheat is also a target staple food crop for biofortification and fortification worldwide. The development of Fe biofortified wheat lines is a strategy to improve the bioavailable long term dietary Fe supply to populations with higher risk of Fe dietary deficiency. The effects of Fe biofortified wheat flour on the gut microbiota were evaluated during a 6-week trial in a study conducted by Beasley et al. [28]. The authors observed an increase in the abundance of the *Actinobacteria* phylum and a reduction in the abundance of *Firmicutes* and *Proteobacteria* in the Fe biofortified wheat group when compared to the control group. Hence, since there is a positive relationship between the abundance of *Actinobacteria* and the consumption of dietary fiber from legumes (beans), fruits and vegetables [53], these observations suggest a potential beneficial effect of this phylum on overall intestinal health. Further, the observed reduction of *Firmicutes, Proteobacteria, Streptococcus*, and *Escherichia* genera confirms the hypothesis that food biofortification can improve the gut health and may reduce the abundance of pathogenic bacterial taxa.

Previously, the *Proteobacteria* phylum was associated with intestinal dysbiosis and inflammatory diseases, such as metabolic disorders and inflammatory bowel disease [54,55]. The Fe biofortified wheat flour contributed to the increased abundance of bacterial populations with recognized probiotic functions, specifically *Bifidobacterium* and *Lactobacillus*, that are able to maintain a symbiotic relationship with the host and to increase the production of SCFAs such as acetic, propionic and valeric acids, and by positively regulating the enzymes of glycolysis and gluconeogenesis [28]. In addition, improvements in dietary Fe bioavailability and physiological status were observed in the biofortified group relative to the control, indicating on a high potential of this food matrix to improve intestinal health.

The protective effects of Fe and Zn biofortified foods on gut microbiota were also evident by the intestinal morphometric evaluation. This association was performed in three of the research manuscripts that were evaluated. One study observed an increase in goblet cell density (per 10 villi) in the group that was fed the Zn biofortified food [31], one study observed an increase in goblet cell number in the group that was fed the Fe biofortified food [28], and one study observed an increase in villi height and diameter, and no difference in goblet cells number in the Fe biofortified group relative to control [15]. In addition, one manuscript reported a depletion in transcription-related proteins and mineral absorption according to the Kyoto Encyclopedia of Genes and Genomes (KEGG) pathways in the Fe biofortified group compared to the control, suggesting that the luminal Fe was not used by the bacteria [30]. However, in four manuscripts the Fe and Zn biofortified foods affected the microbiome by leading towards increased abundance and capacity of intestinal resident bacteria to provide beneficial SCFAs and therefore, favor mineral absorption by the host [15,28,29,31]. Both Fe and Zn are essential micronutrients that are required by beneficial bacteria that make up the gut microbiota. The goblet cells differentiation process is controlled by extrinsic and intrinsic factors, and a healthy gut microbiota increases goblet cells density, which synthesize and secrete the mucus that coats the intestinal lumen. The mucus layer is rich in polysaccharide/protein that protects epithelial cells from the growth of pathogenic bacteria and modifies the luminal environment to favor the absorption of micronutrients [56,57].

The body of evidence reported here indicates that biofortified foods act through beneficial modulation of bacterial taxa, with no adverse risk to the composition of gut microbiota.

### 4.2. Dosages and Reporting Quality

Despite the varied offered dosage, the in vivo studies are based on the analysis of personal food consumption and dietary patterns of relevant populations. Only one study [15] included the source of the database (country dietary survey) that was used to calculate the dietary content and level of the ingredients that were used, including the assessed biofortified food. Studies using Fe biofortified foods were used in a proportion of 34.6–80% from diet and provided between 26.9 and 48.7 μg Fe/g of diet [15,28,30], with no adverse effects on the composition nor genetic capacity of the gut microbiota. Only one study that evaluated zinc biofortified wheat used 75% wheat-based diet, and included 46.5 μg Zn/g of diet [31], with no adverse effects on the gut microbiota composition. Thus, the related microbiome results indicate a promising effect of biofortified foods, with beneficial modulation effect of the host’s gut microbiota.

The current systematic review examines the effects of biofortified foods on gut microbiota. The selection of literature was performed on widely recommended and approved practices for systematic reviews. The risk of bias was verified using the SYRCLE RoB tool [25] and the ARRIVE guidelines [27], aimed to investigate and confirm all possible factors that influence the quality of the in vivo studies that are included in this review. Furthermore, the random sequence generation, the blinding of the investigators, the random of the outcome assessment, and the blinding of the outcome assessor may present potential limitations in some in vivo studies [58,59]. Also, according to ARRIVE guidelines and SYRCLE’S risk of bias tool, to classify studies as “low risk of bias” must be considered and conducted carefully and appropriately.

The number of studies included in this review may be a limitation to directly demonstrate the link between the consumption of biofortified foods and the gut microbiota. Although the articles search was made in four of the most important databases, other databases may include more articles that were not selected. In regard to the in vivo studies with the experimental model of *Gallus gallus*, it is known and established that this model is suitable for the evaluation of dietary mineral bioavailability, mineral metabolism and the gut microbiota, however, no animal model provides physiological responses that can be completely extrapolated to the humans. In addition, in in vivo studies in general, animals consume the specific diet that is provided, based on the tested dietary ingredient and as part of controlled environment and specific study design, which is different from humans in qualitative and quantitative terms. The results found in this review serve as a guide for the development of future clinical trials that may clarify the role of biofortified foods in human health, and specifically the effects on the microbiome.

Hence, this paper provides new insights in this field, and highlights the necessity of more experimental studies and clinical trials to evaluate the gut microbiota due to consumption of biofortified foods. The selected studies allow us to observe and discuss the important associations between the consumption of biofortified foods and the gut microbiota, which is an emerging research area in the field of mineral nutrition.

## 5. Conclusions

The biofortification of foods is a strategy that aims to increase the supply of micronutrients, vitamins and minerals to diverse populations. However, the study of the effects of these foods on the intestinal microbiota is critical to further clarify the potential beneficial effects on host’ intestinal functionality and overall health. Despite the high interindividual variability in the microbiota composition, experimental results showed that the consumption of Fe and Zn biofortified foods modifies the local microbial ecology, increases the abundance of SFCAs producing bacteria and decreases the abundance of potentially pathogenic bacteria, such as *Streptococcus*, *Escherichia,* and *Enterobacter*. This review supports the prospective use of Fe and Zn biofortified foods to increase the colonization of the microbial taxa with beneficial bacteria and therefore to potentially improve the host’s intestinal health. A potential benefit on gut microbiota was verified with the consumption of about 50% biofortified material-based diet. Further studies are needed to strengthen the evidence found in this systematic review, to confirm the amount of the biofortified foods which, in fact, presents a beneficial effect on the gut microbiota in animal model and in humans, and to develop public health strategies to strengthen biofortification programs and encourage the consumption of these foods worldwide.

## Figures and Tables

**Table 1:** Participants, interventions, comparisons, outcomes, and study design (PICOS) criteria for inclusion and exclusion of studies.

Parameter	Inclusion Criteria	Exclusion Criteria
Population	In vivo animal studies	Human studies; in vitro studies; pregnancy and lactation; pathologies different from obesity and micronutrient deficiency
Intervention	Biofortified foods with some micronutrient or their fractions (e.g., flour, soluble extracts)	Do not correlate biofortified foods and gut microbiota; ultra-processed foods; biofortification with compounds different from vitamins and minerals; supplementation
Comparison	Standard foods, or their standard fractions; standard diet for rodents, with no biofortified foods	No control group
Outcomes	Modulation of the health gut microbiota and decrease of pathogenic bacteria	
Study design	Experimental placebo-controlled studies	In vitro studies; reviews; consensus papers; letters to editor; books; book chapters; theses and dissertations; non- animal studies; studies with more than 10 years from publication date

**Table 2:** Characteristics of the eligible studies assessed for Fe and Zn biofortified foods in the gut microbiota modulation.

Reference	Animal Model	Sex/Age	Number of Animals	Type of Food to Intervention	Method of Administration	Duration of Intervention (wk)
Reed et al., 2018 [31]	Cornish Cross broiler (*Gallus gallus*)	Male and female/Hatchlings	30 (*n* = 15 per group)	Zinc biofortified wheat (*Triticum aestivum*)	Oral (in diet)	6
Reed et al., 2017 [30]	Cornish Cross broiler (*Gallus gallus*)	Male and female/Hatchlings	28 (*n* = 14 per group)	Iron biofortified carioca bean (*Phaseolus vulgaris* L.)	Oral (in diet)	6
Dias et al., 2018 [15]	Cornish Cross broiler (*Gallus gallus*)	Male and female/Hatchlings	28 (*n* = 14 per group)	Iron biofortified carioca bean (*Phaseolus vulgaris* L.)	Oral (in diet)	6
Dias et al., 2019 [29]	Chicken embryos (*Gallus gallus*)	Male and female/Day 17th of embryonic incubation	80 eggs (*n* = 10 per group)	Iron biofortified carioca beans (*Phaseolus vulgaris* L.)	Intra-amniotic administration (1 mL per egg)	17th day to 21st day
Beasley et al., 2020 [28]	1st experiment: Chicken embryos (*Gallus gallus*) 2nd experiment: Cornish Cross broiler (*Gallus gallus*)	1st experiment: Male and female/ Day 17th of embryonic incubation 2nd experiment: Male and female/Hatchlings	1st experiment: 40 eggs (*n* ≥ 5 per group) 2nd experiment: 30 (*n* = 15 per group)	Iron biofortified wheat (*Triticum aestivum* L.)	1st experiment: Intra-amniotic administration (1 mL per egg) 2nd experiment: Oral (in diet)	1st experiment: 17th day to 21st day 2nd experiment: 6

**Table 3:** Methods and main findings in studies on the use of Fe and Zn biofortified foods in gut microbiota modulation.

Reference	Experimental Groups	Method of Evaluation of the Gut Microbiota	Microbial Activity
**Zn-biofortified food**			
Reed et al., 2018 [31]	CZn: Standard wheat (75% wheat-based diet; 32.8 ± 0.17 µg Zn/g) BZn: Zn biofortified wheat (75% Zn wheat-based diet; 46.5 ± 0.99 µg Zn/g)	16S rRNA gene sequencing	Change in β-diversity between the CZn and BZn groups. ↔ no difference in abundance between *Firmicutes*, *Actinobacteria*, and *Proteobacteria* phyla according taxon-based analysis; ↔ no differences between groups at the genus level, according taxon-based analysis. LEfSe method: ↑ *Lactobacillus reuteri* and members of the *Dorea*, *Clostridiales*, *Ruminococcus* and *Lachnospiraceae* family in BZn group.
**Fe-biofortified foods**			
Reed et al., 2017 [30]	SFe: Fe standard, 34.6% cream seeded carioca bean based diet (33.7 ± 0.80 μg Fe/g) BFe: Fe biofortified bean, 34.6% cream seeded carioca bean based diet (48.7 ± 1.50 μg Fe/g)	16S rRNA gene sequencing	No change in β-diversity between the BFe and SFe groups; no difference in α-diversity between groups. ↑ *Elusimicrobioa* and *Euryarchaeota* phyla; ↑ *Dehalobacteriaceae* and *Enterococcaceae* family; ↑ unclassified *Dehalobacteriaceae* genus in the BFe group. ↓ *Elusimicrobiaceae*, *Methanobacteriaceae*, and *Methanomassiliicoccaceae* family; ↓ unclassified *Elusimicrobiaceae*, *Methanobrevibacter*, *vadinCA11*, and *Enterococcus* genus in the BFe group; LEfSe method: ↑ *Proteobacteria* and *Firmicutes*; ↓ *Elusimicrobiota* and *Euryarchaeota* at phylum level; ↑ *Campylobacterales*; ↓ *Enterobacteriales*, *Elusimicrobiales*, *Bacteroidales* and E2 at order level; ↑ *Helicobacteraceae*, *Dehalobacteriaceae*, and *Streptococcaceae*; ↓ *Enterobacteriaceae*, *Enterococcaceae*, *Elusimicrobiaceae*, *Coriobacteriaceae*, *Methanomassiliicoccaceae*, and *Methanobacteriaceae* at family level; ↑ *Helicobacter*, *Ruminococcus*, *Coprococcus*, and *Streptococcus;* ↓ *Lachnospira*, *Enterococcus*, *vadinCA11*, *Methanobacterium*, and *Methanobrevibacter* at genus level; ↑ OTUs enriched *Faecalibacterium prausnitzii*, *Barnesiella viscericola*, *Enterococcus cecorum*, and *vadinCA11* in the BFe group.
Dias et al., 2018 [15]	SC: Fe-standard carioca bean-based diet, 42% BRS Perola bean-based diet (40.47 ± 1.84 μg Fe/g) BC: Fe-biofortified carioca bean-based diet, 42% BRS Cometa bean (47.04 ± 1.52 μg Fe/g)	16S rRNA gene sequencing	Change in β-diversity between the BFe and SFe groups; no difference in α-diversity between groups; ↔ no significant differences between groups at the genus level; LEfSe method: Predominance of SCFA-producing Firmicutes in BC group; ↑ *Eggerthella lenta* and *Clostridium piliforme*; members of the *Coriobacteriaceae*, *Dehalobacteriaceae* and *Lachnospiraceae* in the BC group.
Dias et al., 2019 [29]	Non-injected 18 MΩH_2_O Inulin (40 mg/mL) Perola bean extract (Fe standard carioca bean, 3.2 ± 1.5 μg Fe/g) Cometa bean extract (Fe biofortified carioca bean, 1.8 ± 1.1 μg Fe/g) * Esteio bean extract (Fe standard black bean, 1.1 ± 0.6 μg Fe/g) * SMN 39 bean extract (Fe biofortified black bean, 2.2 ± 0.7 μg Fe/g) * Artico bean extract (Fe standard white bean,) * 6.0 ± 1.1 μg Fe/g	PCR amplification of bacterial 16S rDNA for *Lactobacillus*, *Bifidobacterium*, *Clostridium* and *E. coli*	↓ relative abundance of *Bifidobacterium* in biofortified carioca bean extract compared to standard; ↓ relative abundance of *E. coli* in biofortified carioca bean extract compared to standard; ↑ relative abundance of *Lactobacillus* in biofortified black bean extract compared to standard; ↑ relative abundance of *Clostridium* and *E. coli* in biofortified black bean extract compared to standard; ↔ relative abundance of *Lactobacillus* and *Clostridium* in biofortified carioca bean extract compared to standard; ↔ relative abundance of *Bifidobacterium* in biofortified black bean extract compared to standard.
Beasley et al., 2020 [28]	1st experiment: NI: non-injected H_2_O: 18 MΩH_2_O Fe: Fe solution (1 mg/mL) Fe-EDTA: Fe-EDTA solution (77 μM Fe) Fe-NA: Fe-Nicotinamine solution (1.6 mM) C WF: Control wheat flour extract * (0.91 μg Fe/g of extract) B WF: Fe biofortified wheat flour extract * (0.82 μg Fe/g of extract) * 50 mg/mL 2nd experiment: Control: Fe-standard wheat, 80% wheat based diet (25.9 ± 0.12 μg Fe/g) Biofortified: Fe-biofortified wheat, 80% Fe wheat-based diet (28.9 ± 0.13 μg Fe/g)	1st experiment: PCR amplification of bacterial 16S rDNA for *Lactobacillus, Bifidobacterium, Escherichia* and *Clostridium* 2nd experiment: 16S rRNA gene sequencing	1st experiment: ↔ relative abundance of *Bifidobacterium, Lactobacillus, Escherichia* and *Clostridium* in biofortified wheat flour extract compared to the Control. 2nd experiment: Change in β-diversity and α-diversity between the Control and Biofortified groups; ↑ 1.9-fold the proportion of *Actinobacteria*; ↓ 1.2- and 2.0-fold, respectively, the proportion of *Firmicutes* and *Proteobacteria* in ‘Biofortified’ relative to ‘Control’ group at phyla level; ↑ 1.9- and 1.5-fold, respectively, the proportion of *Bifidobacterium* and *Lactobacillus*; ↑ abundance of *Enterococcus*; ↓ proportion of *Streptococcus* (1.7-fold), *Coprococcus* (1.4-fold), *Ruminococcus* (1.2-fold) *Faecalibacterium* (2-fold), and *Escherichia* (2-fold); ↓ *Dorea* abundance in ‘Biofortified’ relative to ‘Control’ group at genera level; ↓ 1.7-fold the proportion of *Lachnospiraceae* and ↑ abundance of *Enterococcaceae* families in ‘Biofortified’ relative to ‘Control’ group.

↔ no change; ↑ increased; ↓ reduced; LEfSe: linear discriminant analysis effect size.

## Data Availability

The data analyzed in this study are openly available in reference numbers [15,28,29,30,31].

## Supplementary Materials

The following is available online at https://www.mdpi.com/2072-6643/13/1/189/s1, Table S1: Risk of bias from experimental studies.
