# Supplementary material for: Effects of Iron and Zinc Biofortified Foods on Gut Microbiota In Vivo (Gallus gallus): A Systematic Review
Source: Nutrients. 2021 Jan 9;13(1):189. doi: 10.3390/nu13010189 (PMC7827887; doi:10.3390/nu13010189)
Supplement: Supplementary file 1 [file nutrients-13-00189-s001.pdf]

Supplementary Material

Table S1. Risk of bias from experimental studies.

Reference	Reed et al., (2018)	Reed et al., (2017)	Dias et al., (2018)	Dias et al., (2019)	Beasley et al., (2020)	Percentage (%)
(1) Title	1	1	1	1	1	100
(2) Abstract	1	1	1	1	1	100
Introduction						
(3) Background information	1	0	1	1	1	80
(4) Primary and secondary objectives	1	1	1	1	1	100
Methods						
(5) Ethical statement	1	1	1	1	1	100
(6) Study design	1	1	1	1	1	100
(7) Experimental procedures	1	1	1	1	1	100
(8) Experimental animals detail	1	1	1	1	1	100
(9) Housing and husbandry conditions	1	1	1	1	1	100
(10) Sample size	0	0	0	0	0	0
(11) Allocating animals to experimental groups	1	1	1	1	1	100
(12) Experimental outcomes	1	1	1	1	1	100
(13) Statistical methods	1	1	1	1	1	100
Results						
(14) Baseline data	1	1	1	1	1	100
(15) Number of animals analyzed	0	0	0	0	1	20
(16) Outcomes and estimation	1	1	1	1	1	100
(17) Adverse events	1	1	1	0	1	80
Discussion						
(18) Interpretation/ scientific implications/study limitations	1	1	1	1	1	100
(19)Generalizability/translation/ relevance to human biology	1	1	1	0	1	80
(20) Funding	0	1	1	1	0	60

0: not reported; 1: reported.
